# The Use of Sensors to Detect Anxiety for In-the-Moment Intervention: Scoping Review

**DOI:** 10.2196/42611

**Published:** 2023-02-02

**Authors:** Rosie Dobson, Linwei Lily Li, Katie Garner, Taria Tane, Judith McCool, Robyn Whittaker

**Affiliations:** 1 National Institute for Health Innovation University of Auckland Auckland New Zealand; 2 Institute for Innovation and Improvement Te Whatu Ora Waitematā Auckland New Zealand; 3 School of Population Health University of Auckland Auckland New Zealand

**Keywords:** anxiety, wearables, sensors, mental health, digital mental health, digital health intervention, wearable device

## Abstract

**Background:**

With anxiety a growing issue and barriers to accessing support services, there is a need for innovative solutions to provide early intervention. In-the-moment interventions support individuals to recognize early signs of distress and use coping mechanisms to prevent or manage this distress. There is potential for wearable sensors linked to an individual’s mobile phone to provide in-the-moment support tailored to individual needs and physiological responses.

**Objective:**

The aim of this scoping review is to examine the role of sensors in detecting the physiological signs of anxiety to initiate and direct interventions for its management.

**Methods:**

Relevant studies were identified through searches conducted in Embase, MEDLINE, APA PsycINFO, ProQuest, and Scopus. Studies were identified if they were conducted with people with stress or anxiety or at risk of anxiety and included a wearable sensor providing real-time data for in-the-moment management of anxiety.

**Results:**

Of the 1087 studies identified, 11 studies were included in the review, including 5 randomized controlled trials and 6 pilot or pretesting studies. The results showed that most studies successfully demonstrated improvements in their target variables. This included overall anxiety and stress levels, and the implementation of in-the-moment stress and anxiety management techniques such as diaphragmatic breathing. There was wide variation in the types of sensors used, physiological measures, and sensor-linked interventions.

**Conclusions:**

This review indicates that sensors are potentially a useful tool in detecting anxiety and facilitating the implementation of a known control mechanism to reduce anxiety and improve mood, but further work is needed to understand the acceptability and effectiveness of this type of intervention.

## Introduction

Anxiety is a critical issue internationally [1], with COVID-19 and its associated lockdowns further increasing global psychological morbidity [2-4]. Stress and anxiety increase the risk of several harmful behaviors including problem drinking, drug use, self-harm, and suicide [5], and the annual cost of the burden of serious mental illness, addiction, and suicide is considerable [6,7].

Access to treatments for anxiety and other mental health conditions are a significant challenge [8]. The current system provides accessible treatment for those with diagnosed serious mental illness but fails to support what is commonly referred to as the *missing middle*, people with mild to moderate mental health needs that may not fit diagnostic criteria. For example, young people in particular generally only access mental health services when their condition has started to significantly affect their daily life, with many continuing to suffer from extended periods of mild to moderate anxiety-related morbidity alone [9,10]. The stigma associated with mental health conditions combined with a perceived lack of accessible support can exacerbate the experience of anxiety for some [11,12]. Even with the growth in web-based/phone services offering immediate support for people with mental health concerns, uptake is varied [13]. Concerns around privacy and being too intrusive are known to prevent people from accessing digital mental health services [14]. Further, facilitators to engagement with these types of interventions include personalization and that they enhance feelings of control over the person’s health [15]. Self-help–focused in-the-moment digital solutions that help individuals to control their own mood and emotions may have the most potential [13].

Wearable devices, which are worn on the body or clothing, provide an innovative tool for the detection, diagnosis, and management of health conditions through the noninvasive measurement of physiological information in real time [8]. There is growing interest in and accessibility of wearable sensors, with rapid uptake in the wider population [16]. Newer wearable sensors enable additional data collection that can be linked to an individual’s mobile phone to tailor support to their needs and physiological responses. The use of digital biofeedback techniques in anxiety is growing [17], presenting the potential for simple personal mobile interventions designed to target anxiety. For example, by alerting individuals early to the signs of their increasing stress or anxiety, there is the potential to circumvent an episode before it worsens. Early evidence has shown the potential for the use of wearable sensors for this purpose [18-22]. Although commercially available sensors are more commonly used for fitness tracking rather than mental health, with their wide uptake, they provide a potential tool for accessible population-based mental health interventions.

Despite the subjective nature of assessing anxiety, the measurement of the physiological changes common in anxiety can be used as indicators of a person’s stress response and experience of anxiety. A number of biomarkers have been found to provide real-time objective measurement of the physiological impacts of anxiety and exposure to stressors [23,24], including cardiac changes (eg, heart rate variability [HRV], pulse photoplethysmography [PPG]), changes in respiratory rate, changes in electrical activity in the brain (eg, electroencephalogram [EEG]), changes in body temperature, and galvanic skin response (eg, electrodermal activity [EDA]). Many of these biomarkers can be measured in real time using commercially available sensors potentially making the detection of anxiety in real-world settings feasible.

This paper provides the findings of a scoping review on the role of sensors in the management of anxiety with the goal of informing the development of digital interventions to treat mild to moderate anxiety.

## Methods

### Design

The aim of this scoping review was to examine the role of sensors in detecting the physiological signs of anxiety to initiate and direct interventions for its management. The review follows the PRISMA-ScR (Preferred Reporting Items for Systematic Reviews and Meta-Analyses Extension for Scoping Reviews) guidelines for the reporting of scoping reviews [25] (see Multimedia Appendix 1), and the protocol was not published.

### Eligibility Criteria

Eligible studies included human participants of any age with anxiety or stress, or at risk of anxiety. Due to the limited number of studies, a decision was made to include studies of participants with stress, not just anxiety. Comorbidity of anxiety with other psychiatric conditions (eg, depression) were also considered since anxiety may often coexist with or be the result of other psychiatric conditions. Eligible studies needed to include a wearable sensor that provided real-time data/information for in-the-moment management of anxiety. As the focus of the review was on the use of sensors for the identification of early signs of anxiety, before the individual is likely to be consciously aware of the anxiety episode, only sensors that collected physiological data in a passive manner (not requiring active engagement from the user) were included; sensors that required manual input of data only were excluded. Sensors had be worn on the body, but no other restrictions on the type were applied (eg, commercial clinical grade). Studies including multifaceted interventions in which sensors were just one component were included in the review.

All study designs were included with the exception of protocols and reviews. There was no limit on year of publication because of the recency of the study subject. No restrictions on comparator or outcome measures were included. The review was restricted to full-text articles published in peer-reviewed journals. Studies were excluded if published in languages other than English or were published only in the form of conference abstracts.

### Study Search Strategy

Searches were conducted from inception through to December 8, 2021, using Embase, MEDLINE, APA PsycINFO, ProQuest, and Scopus. Searches were limited to papers published in the English language. The initial search strategy conducted included words associated with anxiety, such as “stress,” to form search strings. However, these produced results that were too broad, and the term was excluded. An example of the revised search strategy can be seen in Textbox 1. Reference lists of relevant previous reviews and included studies were searched for additional papers.

**Example search strategy.**

1. exp wearable devices/ or wearable*.mp.
2. sensor.mp.
3. 1 or 2
4. exp anxiety/ or anxiety.mp.
5. (psychological stress or psychological distress).mp.
6. 4 or 5
7. 3 and 6
8. limit 7 to English language

### Study Selection

Assessment of study eligibility was performed on the Rayyan platform. The first assessment was screened independently by LLL, identifying duplicates and filtering based on the title and abstract. The second screening of full-text papers was then undertaken by three authors LLL, RW, and RD, and disagreements between reviewers were resolved by discussion.

### Data Extraction

Data were extracted using structured forms including study design (design, duration, setting), population characteristics, intervention type, sensor characteristics (type of sensor, testing conditions, role of sensor, sensor measures, sensor-linked intervention), and outcomes/findings. A narrative synthesis methodology was used to synthesize the data extracted.

## Results

### Study Selection

**Figure 1:**
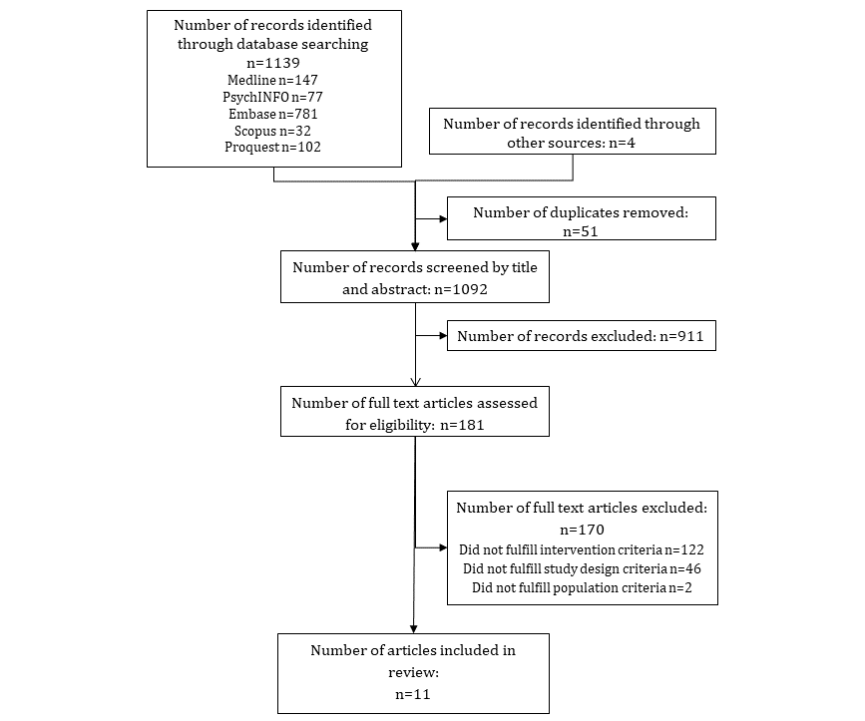
Flow diagram of study selection.

The total number of studies identified was 1139 from database searches, in which 51 were duplicates. A further 4 studies were identified through reference lists. This resulted in 1092 studies screened by title and abstract, with 911 being considered ineligible. The full texts of the remaining 181 full-text papers were reviewed according to the inclusion criteria. At the end of the screening process, 11 articles were included in the scoping systematic review. Figure 1 shows the study selection process, and Table 1 presents a summary of the included studies.

**Table 1:** Characteristics of included studies.

Study	Study design	Duration	Country	Population	Intervention type	Key findings
Chung et al [26], 2021	Pilot study	8 weeks	United States	Adults with a GAD-2^a^ score ≥3 and PHQ-2^b^ score ≤4 aged 24-47 years (N=14)	Assessed the feasibility of an HRVB^c^ wearable device and remote stress management coach to reduce anxiety	Anxiety scores and depression scores decreased over the 8-week intervention**
Crivelli et al [27], 2018	Pretesting study	2 weeks	Italy	Adult professionals with no history of psychiatric or neurological disease (N=16), mean age 44.4 years	To test the training of Vipasyana meditation and technology-mediated mental training for stress management in people who were at risk of stress (which involved real-time acoustic feedback via an app based on changes in the physiological signature of the participant’s mindset)	Significant decrease to perceived stress scores*, situational anxiety**, and anger and fatigue**
Jaramillo-Quintanar et al [28], 2020	Pretesting study	1 session	Mexico	Children with high stress levels (N=29), mean age 8.7 years	To test the feasibility of i-CARE, which measures HR^d^ and blood oxygenation, and provides visual and auditory biofeedback to learn to regulate symptoms of anxiety	Results demonstrate that i-CARE is effective at inducing relaxation in children with high stress (no *P* value reported)
Kizakevich et al [29], 2019	Pilot study	1 year	United States	Adults with a military background (N=328)	To test the effectiveness of 4 different resilience training techniques each with or without HRV^e^ biofeedback for people with a risk of stress; continuous acquisition of HRV data enables analysis of physiological response to stress and breathing training	No conclusions on the results of effectiveness could be drawn from the data
Millings et al [30], 2015	RCT^f^	4 weeks	United Kingdom	University students with >14 on the PSS-10^g^ and <19 on the Beck Depression Inventory (N=92), mean age 23.7 years	Stress management program and a prototype wearable sensor kit comprising of and ECG^h^ and EEG^i^ sensor; compared stress management program alone, program and sensor, or no intervention groups	Significant reductions in levels of stress in those in the program alone condition*; however, sensors impeded the effectiveness of the program potentially due to technical issues
Nguyen et al [31], 2021	RCT	4 training sessions	Canada	Children and adolescents with ASD^j^ aged 8-18 years (N=28)	Use of anxiety meter and breathing techniques; on the fourth visit participants were randomized to receive feedback on anxiety level or no feedback while completing a stress-eliciting task	The anxiety meter improved awareness of anxiety states, which lead to increased likelihood of initiating calming strategies under stress**
Ponzo et al [32], 2020	RCT	4 weeks	United Kingdom	University students aged 18-25 years with score >14 (stress) or >7 (anxiety) on DASS^k^ (N=262)	Biobase program, mobile app comprising psychoeducational content, mood tracking via EMA^l^, and in-the-moment exercises for stress and anxiety (eg, relaxation); real-time sensor data presented to user via app dashboard	Well-being increased and anxiety decreased during intervention and was sustained 2 weeks after the intervention**
Shruthi et al [33], 2021	Pilot study	2 months	India	Students (N=50), mean age 19 years	Wristband to treat anxiety that provided acupressure to the H7 point on the wrist crease when completing a stressful task vs group wearing Fitbit-like band	Intervention group had lower levels of anxiety compared to the active control group (note, no *P* values reported)
Serino et al [34], 2014	Pilot study	120 seconds or more on the app exercises	Italy	App users (N=68)	App that teaches guided relaxation, 3D biofeedback training, and stress self-tracking to help control respiration rate and therefore HR	The stress management exercises along with the app led to a significant decrease in perceived psychological stress**
Smith et al [35], 2020	RCT	4 weeks	United States	Adults who work in knowledgeable occupations (N=215), mean age 33.2 years	App for stress and anxiety that delivers mindfulness-based breathing from MBSR^m^ and Spire Stone to measure subjective emotional state changes; both provide biofeedback about physiological state; the app provided push notifications	Participants in the treatment group experienced 15.8% fewer negative instances of stress**, 13% fewer instances of distressing symptoms, and 28.2%* fewer days feeling anxious compared to waitlist control**
Winslow et al [36], 2016	RCT	8-10 weeks	United States	Veterans (N=16), mean age 39.8 years; self-reported anger and stress	In-person CBT^n^ + sensor and mobile app; alerted the user through the app when stress was detected and presented with prompts or reminders to engage with stress mitigation techniques	High attrition; significant reduction in anxiety and stress observed between intervention and control*; no difference in depression

^a^GAD-2: Generalized Anxiety Disorder 2-item.

^b^PHQ-2: Patient Health Questionnaire–2.

^c^HRVB: heart rate variability biofeedback.

^d^HR: heart rate.

^e^HRV: heart rate variability.

^f^RCT: randomized controlled trial.

^g^PSS-10: Perceived Stress Scale.

^h^ECG: electrocardiogram.

^i^EEG: electroencephalogram.

^j^ASD: autism spectrum disorder.

^k^DASS: Depression Anxiety Stress Scales.

^l^EMA: ecological momentary assessment.

^m^MBSR: mindfulness-based stress reduction.

^n^CBT: cognitive behavioral therapy.

*P<.05, **P<.01

### Study Design

Of the studies included in this scoping review, 5 were randomized controlled trials (RCTs), and 6 were pilot studies or pretesting studies. Sample sizes ranged from 14 to 328 participants. The duration of the studies varied, with the shortest being one session [28,34] and the longest 8-10 weeks [36]. The conditions in which the sensor was worn or used also varied. Two studies were performed in lab conditions [28,31]. Six studies had participants wear the sensor continuously in everyday life [26,29,30,32,33,35], 1 study included combined conditions where participants wore sensors and attended weekly cognitive behavioral therapy (CBT) sessions [36], 1 study required participants to wear the sensor during meditation practice at home [27], and 1 study did not clearly define their testing conditions [34].

### Participant Characteristics

Studies were conducted in the United Kingdom [30,32], the United States [26,29,35,36], Italy [27,34], Canada [31], Mexico [28], and India [33]. All studies were conducted in adult participants except 2 studies that were conducted with children [28,31]. Two studies included participants with anxiety [26,33] (1 diagnosed using a Generalized Anxiety Disorder 2-item score ≥3 and a Patient Health Questionnaire–2 score ≤4; the other had no specified measure), 4 studies were conducted in participants with no reported stress but may have had risk of stress (eg, had a high stress job or had downloaded a stress app) [27,29,34,35], 1 study included participants with at least mild levels of stress [32], 3 studies included participants experiencing high degrees of stress [28,30,36] (but only 1 [36] study had a clinical diagnosis), and 1 study included participants diagnosed with autism spectrum disorder (ASD) [31].

### Sensor Characteristics

Table 2 presents a summary of the sensor characteristics of the included studies. Of the 11 studies, 3 included upper body sensors (ie, on chest or torso) [26,29,35], 3 used sensors that were wrist worn [32,33,36], 2 used sensors attached to the finger or thumb [28,36], and 1 study had a sensor in a pair of eyeglasses [27]; in 2 studies, there was no detail provided [30,34]. Cardiac activity was the main sensor measure in 8 studies [26,28-34]. Within these studies, heart rate (HR) was obtained by all 8 studies and HRV was calculated in 2 studies [26,29,30]. Other sensor measures included accelerometer data [31,36], EEG activity [27], blood oxygenation [28,36], infrared measurements of facial temperature [28], physical activity [30,35], sleep [32,33], step count [32], water intake [33], respiratory effort [35], PPG, EDA, and body temperature [36].

All sensors had some form of capacity to display their sensor data back to the participant. HRV or HR was visually displayed back to participants in 6 studies [28-30,32-34]. Other studies visually displayed PPG, EDA [32], and respiratory amplitude [35]. During the intervention exercises, participants were able to see their biofeedback as raw numbers, or the data was portrayed in another mode (eg, change in color to correspond to their psychological state) [30,31,35]. For example, in the study with children with ASD, anxiety state was also displayed as a color, where green was calm, yellow was rising anxiety, and red was anxious [31]. Two studies that measured EEG activity provided real-time auditory feedback based on their mindset [27] and alpha waves relative to beta waves [30], which both corresponded to their state of relaxation.

In 3 studies [26,31,35], sensors provided physical feedback to the participants. Two studies used a vibration to alert users of their anxious state [31,35], and 2 studies used vibrations to help guide the user in their breathing in conjunction with visual biofeedback [26,35].

Most studies (10/11, 91%) included sensors that were linked to some form of breathing techniques such as diaphragmatic breathing to help manage stress or anxiety. Eight studies used guided diaphragmatic breathing [26,28-32,34,36], and 2 studies included mindfulness-based breathing techniques [27,35]. Only 1 study did not include any breathing techniques but used acupressure on the wrist to help reduce anxiety [33].

Some of the studies had interventions that included additional information or therapy to supplement stress or anxiety management. Three studies included CBT-based techniques as part of their intervention [26,30,36], and 1 study had participants attend CBT once per week [36]. Two studies included psychoeducation on stress management [30,32]. In terms of whether participants were receiving treatment for anxiety outside the intervention, 1 study excluded participants who were receiving other forms of treatment [26], and the remaining studies did not specify this information.

**Table 2:** Sensor characteristics.

Study	Type of sensor	Testing conditions	Role of sensor	Sensor measures	Sensor-linked intervention	Sensor usability and acceptability
Chung et al [26], 2021	Lief Smart Patch; worn on torso under clothing	Continuous wear in everyday life	Reduce anxiety	HR^a^, HRV^b^, and accelerometer data through continuous monitoring	HRVB^c^ wearable device and remote stress management coach	86% of participants wore the patch and completed ≥1- to 3-min HRVB exercise on at least 40 of 56 days; only 43% completed 3 or more 3-min HRVB exercises on at least 40 of 56 days
Crivelli et al [27], 2018	Lowdown Focus brain-sensing eyeglasses	Wore glasses when participating in meditation practice	Stress management	EEG^d^ activity	Vipasyana meditation and technology-mediated mental training for stress management (which involved real-time acoustic feedback via an app based on changes in physiological signature of the participants’ mindset)	Not reported
Jaramillo-Quintanar et al [28], 2020	i-CARE; sensor on child’s finger	Lab administered	Regulate symptoms of anxiety	HR, blood oxygenation, and infrared measurements of facial temperature	i-CARE, which measures HR and blood oxygenation, and provides visual and auditory biofeedback	Not reported
Kizakevich et al [29], 2019	BART; chest belt sensor	Continuous wear in everyday life	Manage anxiety	HRV	4 different resilience training techniques each with or without HRV biofeedback; continuous acquisition of HRV data enables analysis of physiological response to stress and breathing training	Participants in the 6-week training regimen completed; 600 sessions during the first week; however, over the next several weeks, training compliance fell by almost one-third and to about one-half after 1 month
Millings et al [30], 2015	Prototype wearable sensor kit	Continuous wear in everyday life	Stress management	HRV, HR, and physical activity	Stress management program and a prototype wearable sensor kit comprising of an ECG^e^ and EEG sensor	Qualitative results found that many participants experienced technical issues that caused frustration
Nguyen et al [31], 2021	Wearable Shimmer2 unit	Lab administered	Manage anxiety	HR, anxiety-level feedback on tablet (green: calm; yellow: rising anxiety; red: anxious)	Use of anxiety meter and breathing techniques	Not reported
Ponzo et al [32], 2020	Biobeam; wrist worn; continuous wear	Continuous wear in everyday life	Manage stress and anxiety	Sleep duration and quality, HR, step count	Biobase program, mobile app comprising psychoeducational content, mood tracking via EMA^f^ and in-the-moment exercises (eg, relaxation); real-time sensor data presented to user via app dashboard	Not reported
Shruthi et al [33], 2021	Prototype wrist band	Continuous wear in everyday life	Manage anxiety	Oxygen levels, water intake, sleep, HR	Wrist band that provided acupressure to the H7 point on the wrist crease when completing a stressful task vs group wearing Fitbit-like band	Not reported
Serino et al [34], 2014	App called Positive Technology	Worn when using the app (not well defined)	Manage stress	HR	App that teaches guided relaxation, 3D biofeedback training, and stress self-tracking	Not reported
Smith et al [35], 2020	Device called Spire Stone: a clothing-attached device	Continuous wear in everyday life	Manage stress and anxiety	Respiratory effort and physical activity	App that delivers mindfulness-based breathing from MBSR^g^ and Spire Stone to measure subjective emotional state changes; both provide biofeedback about physiological state; the app provided push notifications	Participants wore the device 52% of the days during the intervention period; 75% completed at least one educational guided breathing session and only 19% completed all 5 sessions
Winslow et al [36], 2016	E3 band, wrist worn	Continuous wear in everyday life	Manage stress	PPG^h^, EDA^i^, body temperature, accelerometer, HR	In-person CBT^j^ + sensor and mobile app; alerted the user through the app when stress was detected and presented with prompts or reminders to engage with stress mitigation techniques	Individuals in the experimental group completed a significantly greater number of therapy sessions compared to the control group; 1 participant in the experimental group did not use the app but completed CBT

^a^HR: heart rate.

^b^HRV: heart rate variability.

^c^HRVB: heart rate variability biofeedback.

^d^EEG: electroencephalogram.

^e^ECG: electrocardiogram.

^f^EMA: ecological momentary assessment.

^g^MBSR: mindfulness-based stress reduction.

^h^PPG: photoplethysmography.

^i^EDA: electrodermal activity.

^j^CBT: cognitive behavioral therapy.

### Study Outcomes

Of the 5 RCT studies included in this review, 4 found significant improvements to measures of stress or anxiety. One study assessed the effectiveness of an anxiety meter to improve awareness of anxiety symptoms and, therefore, engage in relaxation techniques in children with ASD [31]. Researchers found after four visits, the anxiety meter improved awareness of anxiety states, which then led to increased likelihood of engaging in calming strategies under stress. In another study, researchers tested the efficacy of a mobile app called BioBase paired with a wearable device on reducing anxiety and improving well-being [32]. Participants were university students with elevated levels of anxiety and stress. Results showed that well-being improved and anxiety decreased during the intervention compared to waitlist controls, and this was sustained 2 weeks post intervention. Smith et al [35] looked at whether an app that delivers mindfulness-based stress reduction and a sensor could improve mental health outcomes. Participants were employees who were randomized to the intervention or waitlist control. Researchers found that those in the intervention group experienced 15.8% fewer negative instances of stress, 13% fewer instances of distressing symptoms, and 28.2% fewer days feeling anxious post intervention compared to the controls. Lastly, a study assessed the effectiveness of a sensor paired with in-person CBT for stress management in a sample of veterans who were experiencing high levels of stress, finding a significant reduction in stress and anxiety between the intervention group and controls at follow-up [36].

Interestingly, 1 study found contrasting findings [30]. Researchers assessed whether the effectiveness of an online intervention for stress could be enhanced by using a sensor. Students who were stressed were randomized to either the stress management program alone, the program and the sensor, or no intervention. After 4 weeks, the results showed significant reductions in stress in the program alone condition and similar but weaker reductions in the program and sensor condition compared to the controls. Therefore, researchers suggested that it was possible that the sensor impeded the efficacy of the stress management program.

Three pilot or pretesting studies also found promising improvements to either stress or anxiety. One study assessed the effectiveness of an HRV biofeedback wearable device and stress management coach to reduce symptoms of anxiety [26]. Researchers found that after the 8-week duration, the intervention led to a clinically significant decrease in anxiety and depression scores [26]. In another pilot study, researchers tested the effectiveness of an app that teaches guided relaxation, 3D biofeedback training, and stress self-tracking for stress and anxiety. For participants who engaged in 120 seconds or more on the exercises, there were significant decreases in perceived psychological stress and HR; however, the change in HR was not significant [34]. Lastly, Crivelli et al [27] looked at the effectiveness of a training protocol that used brain-sensing eyeglasses to help participants become more aware of their mindset and develop greater stress coping. Researchers found that, post training, there was a significant decrease in stress scores, situational anxiety, anger and fatigue.

## Discussion

This review explored the use of wearable sensors to detect, manage, and treat anxiety. The results showed that most studies successfully demonstrated improvements in their target variables. This included overall anxiety and stress levels, and the implementation of in-the-moment stress and anxiety management techniques such as diaphragmatic breathing. Where applicable, most studies also noted a reduction in secondary symptoms such as depression, anger, and negative emotions. The results therefore indicate that sensors are potentially a useful tool in detecting stress and anxiety and facilitating the implementation of a known control mechanism to reduce anxiety and improve mood.

The utility of sensors was shown to extend beyond sensing physiological signs of anxiety and stress. Sensors have the potential not only to alert the user to their change in emotional state but also to connect with other systems and prompt interventions that can treat the detected changes and deliver those interventions through the sensor itself. For example, the results of this review showed that when a user is alerted to their change in anxiety, the screen on some sensors could be used to guide the user through the subsequent recommended breathing exercise. Exploration of other in-the-moment control mechanism or therapeutic techniques could be explored to diversify and maximize the treatment options available through sensors.

This review also identified that sensors can be used by a wide variety of people in a range of contexts. Studies ranged in age and clinical severity, from mild to moderate levels of stress, depression, or anxiety to diagnosed clinical disorders including anxiety, posttraumatic stress disorder, and ASD. Studies also varied in length and nature, from wearing sensors and learning coping mechanisms in brief laboratory sessions to wearing the sensors for multiple weeks of everyday life. The variety of populations and contexts included in the literature thus far supports the idea that wearable sensors are a broadly applicable tool with flexible utility that may offer benefit to most populations.

This is an initial scoping review to determine the body of existing knowledge on this topic. The limitations of this review are that there are only a small number of RCTs; several studies were only performed in laboratory conditions; we did not limit the review to just anxiety (the inclusion of stress); and the sensors and conditions of use (eg, time, how they were worn) varied considerably. This indicates that there is not yet sufficient evidence to show that these interventions are feasible in real-world situations, although there appears to be sufficient potential for further investigation. With the growing use of consumer-wearable devices it may be possible in the future to use population-based, real-world data in this field, but there is much to be worked out before this can proceed in relation to ethics, privacy, and ownership of data.

There is also substantial further research required on the digital interventions that may be acceptable and effective in the moment for different cohorts, for example, the question of whether the sensor data should be made visible to the individual at the time or just the intervention. There is a risk that the availability of such continuous data could heighten anxiety in some people or could become the focus of their anxiety. It is also not clear whether an intervention based around identifying the physiological signs of anxiety would need to be long-term or could potentially *train* an individual to be more aware of their own symptoms earlier.

The acceptability and usability of the sensors is going to play a key role in their success, as was highlighted by Millings et al [30]. Technical issues can result in frustration and may not only result in disengagement with the intervention but also contribute to an individual’s anxiety. Although there is potential for more work in this area, the authors are aware that the research and development of such interventions should be co-designed with potential end users. Without end-user involvement, interventions could miss the mark in terms of developing effective and acceptable digital health programs.

## Appendix

Multimedia Appendix 1 PRISMA-ScR (Preferred Reporting Items for Systematic Reviews and Meta-Analyses Extension for Scoping Reviews) checklist.

## Abbreviations

ASD: autism spectrum disorder
CBT: cognitive behavioral therapy
EDA: electrodermal activity
EEG: electroencephalogram
HR: heart rate
HRV: heart rate variability
PPG: pulse photoplethysmography
PRISMA-ScR: Preferred Reporting Items for Systematic Reviews and Meta-Analyses Extension for Scoping Reviews
RCT: randomized controlled trial
